# Getting Under the Skin: The Association Between Social Isolation and Inflammatory Markers

**DOI:** 10.1093/geroni/igaa057.1975

**Published:** 2020-12-16

**Authors:** Thomas K M Cudjoe, Carl Latkin, David Roth, Roland Thorpe, Cynthia Boyd

**Affiliations:** 1 Johns Hopkins University School of Medicine, Baltimore, Maryland, United States; 2 Johns Hopkins Bloomberg School of Public Health, Baltimore, Maryland, United States; 3 Johns Hopkins University, Baltimore, Maryland, United States

## Abstract

Social isolation is a risk factor for morbidity and mortality comparable to well-established risk factors including smoking, hypertension, and a sedentary lifestyle. Specific mechanisms that connect social isolation to important health outcomes remain unclear. We examine the cross-sectional relationship between social isolation and two biological markers: Interleukin-6 (IL-6) and C-Reactive Protein (CRP) in a nationally representative population of community dwelling older adults (IL-6: n=4336, CRP: n=4178) from the National Health Aging Trends Study in 2017. Adjusting for age, gender, race, income, tobacco use, body mass index, and multiple chronic conditions, we found that social isolation compared to no social isolation was associated with higher levels of IL-6 (p = 0.043) and CRP (p = 0.038). These results suggest that investigating inflammatory pathways between social isolation and morbidity and mortality is important.

